# From Social to Symbolic: Investigating the Neural Networks Involved in Emoji and Facial Expression Recognition

**DOI:** 10.1007/s10548-025-01163-6

**Published:** 2026-01-20

**Authors:** Alice Mado Proverbio, Lodovica Bacciocchi, Arianna Pavoni

**Affiliations:** 1 Cognitive Electrophysiology Laboratory, Department of Psychology of University of Milano-Bicocca, Milan, Italy; 2 Department of Psychology of University of Milano-Bicocca, Piazza dell’Ateneo nuovo 1, Milan, 20162 Italy

**Keywords:** Social neuroscience, Faces, Symbols, Metalizing, Limbic, Self, medial prefrontal cortex, Neuroimaging, Source reconstruction, LORETA

## Abstract

Facial expressions and emojis serve as fundamental social cues, yet their neural processing remains distinct. Using swLORETA source reconstruction based on EEG/ERP signals at N170 stage, we analyzed participant-specific brain activations in 50 young, healthy individuals performing an emotion recognition task with real faces and emojis. Behavioral results revealed higher accuracy and faster reaction times for emojis compared to faces consistent with prior electrophysiological findings on P300 amplitude effects. Both stimulus types activated bilateral fusiform areas (BA19/37) and the orbitofrontal cortex. Neuroimaging results also showed that only human facial expressions engaged the medial and superior frontal cortex (BA 10, BA 8), involved in mentalization and Theory of Mind, as well as limbic structures such as the left uncus, associated with instinctual emotion processing, and the left fusiform gyrus BA 19, to a much greater extent. Conversely, emoji recognition recruited bilateral temporal cortices, the right inferior frontal gyrus, and superior parietal cortex—regions implicated in symbolic and semantic processing, akin to numerical cognition. This suggests that while faces are processed as biologically and socially relevant stimuli, emojis are interpreted as abstract symbols. Furthermore, greater hemispheric asymmetry in emoji recognition supports distinct cognitive strategies for decoding schematic versus naturalistic expressions. Overall, our findings indicate that facial expression processing relies on socio-affective networks, whereas emoji recognition engages symbolic, linguistic, and sensorimotor circuits, highlighting fundamental differences in how the human brain processes digital versus natural social cues.

## Introduction

Emoji facial symbols are now pervasive in online communication, particularly across instant messaging and social media platforms (Tieu et al., 2024), as they enhance the comprehension of indirect speech (Hancock et al. 2024) and facilitate the transmission of emotional meaning, thereby shaping social interactions (Weiß et al. 2019, 2024). Neuroscientists have sought to elucidate the similarities and differences between the two socio-affective communication systems, investigating which types of messages each conveys most effectively, their respective processing dynamics, the underlying neural mechanisms, and their distinctive functional properties. Event-related potential (ERP) studies comparing face and emoji processing have revealed that happy, disgusted, and sad emojis are recognized more accurately than fearful ones, while happy and angry facial expressions are more readily distinguished than fearful faces. Fear, therefore, appears difficult to decode across both formats. In these paradigms, incongruently primed emojis and faces elicit larger N400 responses, whereas the P300 component exhibits the opposite trend. Weiß et al. (2020) showed that emojis elicit more negative N170 and N2 potentials than human faces, suggesting differential early visual and cognitive processing. Conversely, Liao et al. (2021) reported superior recognition accuracy and faster reaction times for facial expressions—specifically in pain-related contexts—accompanied by comparable P2 and LPP amplitudes but diverging P3 and N2 patterns: P3 was more sensitive to facial pain, while N2 was stronger for pain in emojis.

Collectively, these findings indicate that emojis—owing to their schematic, unambiguous, and identity-independent nature—can sometimes be comprehended more efficiently than real faces (Dalle Nogare et al. 2023). Such evidence supports the notion that emojis may function as a standardized and effective form of nonverbal communication in textual exchanges. Extending this perspective, a combined EEG and source reconstruction (LORETA) study by Dalle Nogare and Proverbio (2023) demonstrated that the emoji-related N170 (150–190 ms) was sensitive to affective valence, much like the face-related N170 (Weiß et al. 2020). However, its neural generators were localized not in the fusiform face area (FFA) but predominantly in the occipital face area (OFA) and in object-related cortical regions. Both faces and emojis also activated the limbic system and orbitofrontal cortex—areas implicated in affective comprehension of visual information (Shamay-Tsoory et al. 2006; Leopold et al. 2012)—supporting the idea that anthropomorphization mechanisms underlie the processing of schematic social cues. The prominent involvement of the OFA, rather than the FFA, aligns with the interpretation that emojis, as highly stylized visual representations, primarily engage early visual and object-recognition networks. More recently, Dalski et al. (2024) reported that the mid and posterior occipito-temporal sulci exhibited greater sensitivity to emojis than to words, suggesting that these regions may be tuned to pictorial or symbolic representations and contribute to visual-semantic integration and the rapid detection of socially salient cues.

Despite these converging findings, the precise neural mechanisms underlying the emotional and cognitive interpretation of social information conveyed by real faces versus schematic surrogates remain only partially understood. While both efficiently communicate affective states, it is plausible that mentalizing processes, particularly those related to attributing beliefs and intentions, i.e., a Theory of Mind (ToM; Premack and Dasser 1991), are more strongly engaged when perceiving real human faces than symbolic depictions. In the present study, we adopt a broad operational definition of ToM, interpreting it as the capacity to attribute to others beliefs, intentions, but also desires and emotions (i.e., a higher-order mental state attribution), rather than limiting it to simple facial emotion recognition. As described by Byom and Mutlu (2013), mentalizing involves the complex inferential reasoning about other minds that underlies social interactions. The theoretical chain underlying our paradigm is as follows: when participants engage in passive viewing of real human facial expressions, they are exposed to subtle cues of another person’s internal state (musculature, gaze, expression dynamics). These cues automatically trigger perceptual-affective pathways (face recognition, emotional resonance) which, in turn, afford spontaneous inferential processes concerning that person’s mental states (what they might be thinking, intending or feeling). Empirical evidence supports a link between face-processing and affective mentalizing: for example, impaired face recognition predicts poorer “affective ToM” performance (Altschuler et al. 2021). Therefore, we hypothesize that real faces, by virtue of their richer, variable and socially embedded expressive content, will provoke more robust engagement of mentalizing neural circuits (e.g., medial/superior prefrontal cortex) than simplified symbolic surrogates such as emojis, which may suffice for basic emotion recognition but less so for full mental-state attribution.

To directly address this issue, the present study investigated the neural underpinnings of these processes in a cohort of 50 individuals, employing an analytical framework of single case neuroimage analysis designed to prevent the attenuation of fine-scale neural activations due to mediation/averaging effects in the group data. In fact, the application of source reconstruction to group (average event-related potentials, or ERP grand means) instead of individual data might reduce or eliminate information about sophisticated neural circuits showing a large inter-individual variability. We specifically hypothesized that if mentalization, ToM and social attributions played a more prominent and salient role during real face perception, this would be reflected in greater engagement of the medial/superior prefrontal cortex (BA10) in this process (Sommer et al. 2007; Stuss et al. 2001; Hirao et al. 2008; Hartwright et al. 2014), compared to emoji processing. To the best of our knowledge, no direct studies have yet compared ToM processing between real faces and emojis. However, existing research has investigated how the brain interprets emojis relative to real facial expressions. Notably, studies suggest that the brain processes emojis with remarkable efficiency, often recognizing them more rapidly and accurately than real facial expressions, particularly for negative emotions such as fear, anger, and disgust. This finding implies that the schematic and standardized nature of emojis may facilitate emotional recognition more effectively than the variability inherent in real faces. Furthermore, while emojis are perceived as facial representations, the neural mechanisms underlying their interpretation appear to differ from those engaged during real face perception. Specifically, core face-processing regions, such as the fusiform face area (FFA) and superior temporal sulcus (STS), do not exhibit the same activation patterns in response to emoji stimuli, indicating distinct processing pathways (Dalle Nogare and Proverbio 2023). Nevertheless, Churches et al. (2014) demonstrated that both faces and emoticons elicit activity in the occipito-temporal cortex, suggesting a degree of overlap in their visual processing. However, the extent to which emojis engage higher-order socio-cognitive mechanisms, particularly those implicated in mental state attribution, remains unclear. Although these findings do not directly examine ToM-related differences between real faces and emojis, they suggest that the neural circuits subserving social cognition may be differentially engaged by these stimuli. Given that ToM involves the attribution of beliefs, intentions, and emotions to others, it is plausible that these processes were more robustly activated while deciphering real human facial expressions—especially in the medial prefrontal cortex (BA10)—which convey richer and more nuanced social and affective cues compared to the simplified, symbolic nature of emojis.

## Materials and Methods

### Participants

Twenty-five people (12 females and 13 males) aged 18 to 35 years (mean age = 22.16, SD = 2.48) participated in the face study. The current sample size was tested for power analysis with alpha level = 0.05. They were all right- handed, as assessed through the administration of the Edinburgh Handedness Inventory (mean score = 0.82). Further twenty-six people (13 females and 13 males) aged 18 to 35 years (mean age = 22.35, SD = 2.6) participated in the emoji study. They were all right- handed, as determined by the Edinburgh Inventory (mean score = 0.83). The data of 1 male participant was discarded for excessive EOG artifacts.

All 50 participants were University students and earned 0.6 academic credits for their participation. They all declared to have never suffered from psychiatric or neurological deficits and to have good or corrected-to-good vision. Before taking part in the experiment, participants signed written informed consent forms. The study was carried out in accordance with the relevant guidelines and regulations and was approved by the ethics committee (CRIP, protocol number RM-2021-401) of University of Milano-Bicocca.

## Stimuli

**Fig. 1:**
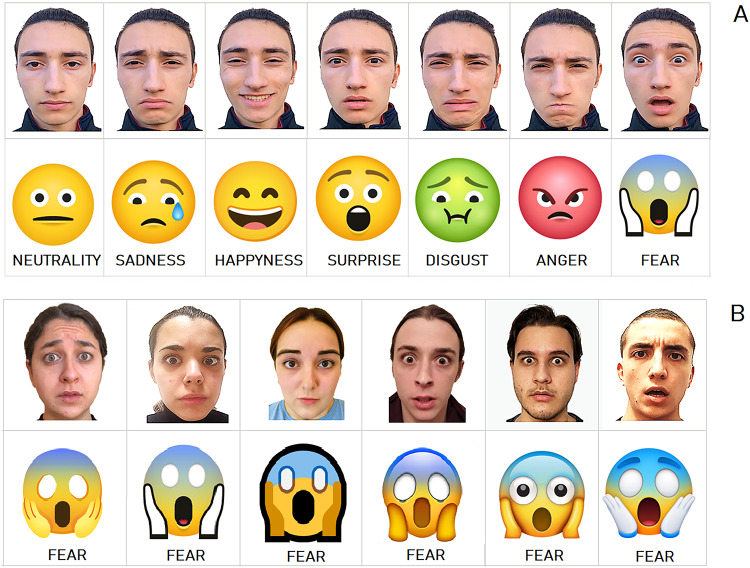
Examples of facial expressions and corresponding emoji representations of basic emotions. **(A)** Series of male facial expressions depicting seven fundamental emotions (neutrality, sadness, happiness, surprise, disgust, anger, and fear) paired with their commonly used emoji counterparts. **(B)** Series of facial expressions from different individuals, all depicting the same emotion (fear), shown alongside emojis rendered in varying graphic styles to simulate inter-individual identity differences in visual representation

This study employed 96 distinct visual stimuli, including 48 human facial expressions and 48 emoji images, each depicting 8 variations of 6 universal emotions (Ekman 2003)—happiness, sadness, surprise, fear, anger, and disgust—plus a neutral expression (See Fig. 1A). Eight different individuals (4 female, 4 male) posed for the facial expression set, providing audiovisual consent. Emoji icons were obtained from free web platforms and depicted the same emotions across eight distinct graphic styles, to emulate inter-individual differences in facial appearance and identity.

*Faces*. Ten Caucasian university students (5 males, 5 females; mean age ≈ 23 years) served as models for the photographic stimuli. Participants were instructed not to wear any accessories (e.g., earrings, glasses, makeup, hair ornaments, necklaces, tattoos, or piercings) and to be clean-shaven. All wore a black shirt and tied their hair back behind the ears. The upper dark region above the forehead generated by the hairline was retained to preserve the natural human appearance of the faces, while minimizing visual distractions. Each actor was asked to portray seven emotional expressions by vividly recalling specific autobiographical episodes through the Stanislavsky method, thereby eliciting spontaneous and genuine facial expressions rather than artificial mimicry detached from the emotional state. A positive scenario was encouraged for the expression of surprise. All models provided written informed consent and signed a privacy release form.

The resulting set included 56 photographs (8 actors × 7 emotions), which were validated on a separate sample of 50 students (25 females, 24 males, 1 gender-fluid participant; mean age 23.7 years) with normal or corrected-to-normal vision and no history of neurological or psychiatric disorders (Dalle Nogare et al. 2023). Participants were presented with randomized sequences of stimuli, one at a time, centered on the screen, and were instructed to select as accurately and rapidly as possible the emotion label best describing each facial expression. The overall recognition accuracy was 87.35%, consistent with prior benchmarks (e.g., Carbon 2020). Specifically, hit rates were 98.5% for happiness, 86.7% for surprise, 80.1% for sadness, 89.3% for anger, 72.7% for fear, 86.0% for disgust, and 98.2% for neutrality. To control for physical appearance, an additional group of 12 students (7 females, 5 males; aged 18–25 years) rated the neutral faces for attractiveness on a 3-point Likert scale (1 = “not attractive”, 2 = “average”, 3 = “attractive”). No gender differences were observed (mean score = 1.83 for females, 1.82 for males), confirming that the actors were perceived as moderately attractive, thus representative of a normal-looking population. Final face images measured 3.37 × 5 cm (3°22′ × 5°).

*Emojis.* A corresponding set of emoji stimuli was drawn from freely available web platforms (Apple, Google, Microsoft, WhatsApp, Twitter, Facebook, Joypixels, and Icons8; accessed April 1, 2021). Emojis represented the same seven emotional categories as the facial photographs. For each emotion, eight stylistic variants were included, for a total of 56 emojis (see some example in Fig. 1B). All images were standardized for size (4 cm in diameter) and mean luminance, and were slightly adjusted to equalize average luminance, color saturation, and overall balance. The selection of stimuli was based on a preliminary behavioral validation conducted online via Google Forms on a separate group of 48 students (24 males, 24 females; mean age ≈ 23 years). Participants viewed individual emojis in random order and were asked to select, as accurately and quickly as possible, the emotion label best matching the displayed expression. From an initial pool of 168 emojis (24 styles × 7 emotions), the eight sets achieving the highest recognition accuracy (totaling 56 emojis) were retained as experimental stimuli. The mean recognition rate for the final set was 79.40%, with emotion-specific accuracies of 94.53% for happiness, 76.04% for surprise, 84.12% for sadness, 96.09% for anger, 57.29% for fear, 91.15% for disgust, and 56.51% for neutrality.

Stimuli—either facial expressions or emojis, depending on the experimental group—were presented against an isoluminant gray background at the center of a PC screen positioned 100 cm from the participant’s eyes. Participants were comfortably seated in an electrically and acoustically shielded room, and the experiment took place under scotopic lighting conditions. Each stimulus, standardized to a diameter of 8 cm, was equiluminant. Before each stimulus, an emotional word (e.g., *sadness or surprise*, presented in Italian) was displayed in Arial font for 200 ms at the center of the screen. The interstimulus interval (ISI) varied randomly within 300 ± 100 ms, after which the emoji or facial expression appeared for 800 ms. The intertrial interval (ITI) was 700 ± 100 ms. Participants were instructed to quickly and accurately assess whether the facial expression or emoji was emotionally congruent with the preceding word by pressing one of two designated keys.

## EEG Recording and Source Reconstruction

EEG signals were recorded as described in Dalle Nogare and Proverbio (2023) where ERP waveforms were analyzed. Here only signals relative to congruent signals are considered, regardless of emotional expression. EEG data were digitalized from 128 scalp sites (Oostenveld and Praamstra 2001) at a sampling rate of 512 Hz by means of an *EEProbe* recording system (*Advanced Neuro Technology* (ANT) Enschede, The Netherlands). Horizontal and vertical eye movements were also recorded using the linked ears as the reference lead. The EEG and electrooculogram (EOG) were amplified with a half-amplitude bandpass of 0.16–70 Hz. Electrode impedance was maintained below 5 kΩ. EEG epochs were synchronized with the onset of stimulus presentation. Computerized artifact rejection was performed to discard epochs in which eye movements, blinks, excessive muscle potentials or amplifier blocking occurred. The artifact rejection criterion was a peak-to-peak amplitude that exceeded ± 50 µV, which resulted in a rejection rate of ∼5%. To identify the cortical sources of the face-specific activity corresponding to N170 component, individual swLORETA models were conducted for the emoji and face condition in the 150–190 ms time window (following: Rossion and Jacques 2011; Eimer 2011; Dalle Nogare and Proverbio 2023), for a total of 50 swLORETAs. The N170 component was identified in ERP responses elicited by (congruently defined) emojis and faces. The measurement window was centered on the point of maximal negative voltage (i.e., the waveform inflection point) and spanned ± 20 ms around that peak (i.e., a 40-ms window). Accordingly, for both stimulus types, the N170 amplitude was quantified within the 150–190 ms time window. Low-Resolution Electromagnetic Tomography (LORETA) is a powerful source reconstruction technique used in Brain-Computer Interfaces (BCIs) to localize neural activity with high spatial resolution. Utilizing Electroencephalography (EEG) data and a realistic head model with a distributed source model, LORETA avoids the need for restrictive assumptions and efficiently localizes neural sources (Grech et al. 2008). However, its spatial resolution can be limited in the presence of noise, or when multiple dipoles are active simultaneously (Pascual-Marqui, 1994). To address this limitation, Palmero-Soler and colleagues (2007) proposed an improved version called SwLORETA, which incorporates a Singular Value Decomposition based lead field weighting. Additionally, synchronization tomography and coherence tomography based on SwLORETA were introduced to analyze phase synchronization and standard linear coherence, applied to current source density (Palmero-Soler et al. 2007). Palmero-Soler et al. (2007) compared the conventional LORETA (sLORETA) with its weighted variant, SwLORETA, and found that SwLORETA offers significant improvements in several key areas. For instance, localization error—defined as the distance between the peak of the current distribution and the simulated dipole’s true location—decreases with increasing eccentricity, with SwLORETA consistently outperforming sLORETA across all tested eccentricity levels and signal-to-noise ratios (SNR). In addition, activation volume—measured as the number of voxels with a strength exceeding 60% of the maximum current source density (CSD)—is reduced when using SwLORETA because its reconstruction of the CSD is more narrowly concentrated around the actual dipole location. Similarly, activation probability, which is determined by the fraction of instances in which the simulated dipole’s position exceeds 60% of the maximum CSD, nearly reaches its maximum with SwLORETA, further underscoring its superior performance. Collectively, these enhancements underscore SwLORETA’s improved precision in localizing neural sources, thereby boosting the performance of brain–computer interface (BCI) applications. In essence, SwLORETA represents a significant advancement in source reconstruction techniques for BCI, offering enhanced spatial resolution and localization accuracy compared to sLORETA. For each subject and experimental condition, active dipoles were identified and subsequently categorized based on their Talairach coordinates, hemisphere, cerebral region, and Brodmann area (BA). Each active source was then assigned to a specific Brodmann area and treated as a distinct region of interest (ROI). Finally, LORETA solutions were analyzed in terms of both activation strength and the overall frequency of activation observed across the sample.

The strength of activation of electromagnetic dipoles underwent a 2-way repeated-measure ANOVA whose factors of variability were: type of stimulation (Emoji, face) and Cerebral hemisphere (Left, right). Tukey post-hoc comparisons were carried out to test differences among means. Because emoji lack intrinsic gender attributes and gender was not an experimental factor, no analyses involving participant gender or gender interactions were conducted, as subdividing the sample would have substantially reduced statistical power and yielded unreliable effect-size estimates. The effect size for the statistically significant factors was estimated using partial eta squared (η_p_^2^) and the Greenhouse–Geisser correction was applied to account for non-sphericity of the data. All the analyses were performed via *Statistica* software (version 10) by StatSoft. The assumption of sphericity was assessed for the repeated measures ANOVA involving hemisphere. The Greenhouse-Geisser epsilon (ε) value for hemisphere was found to be 1, indicating that the assumption of sphericity was met. Additionally, a two-way repeated-measures ANOVA was conducted to examine the frequency of activation across all regions of interest (ROIs) identified in swLORETA solutions for the entire sample, with stimulation type (Emoji, Face) and cerebral hemisphere (Left, Right) as factors of variability. Furthermore, independent-samples t-tests were performed on the activation frequency of individual ROIs that exhibited large inter-individual variability (≥ 5 units) between the emoji and face processing conditions. To control for multiple comparisons across ROI-level independent-samples *t*-tests, a Benjamini–Hochberg false-discovery rate (FDR) correction was applied (family-wise across all ROIs tested; *q* < 0.05.

## Results

Fifty individual swLORETA solutions were applied to electrical potentials recorded from 128 sites in the time window corresponding to the N170 face-specific component. The occipito-temporal regions with the highest activation magnitude were identified in Brodmann Areas (BA) 18, 19, 21, and 37. Individual mean amplitudes were computed, and the group-level Talairach coordinates were determined for both stimulus types. Additionally, all active dipole activations across the entire brain were examined and quantified, with activation frequencies calculated both globally and across participants. ANOVA and t-tests were conducted to assess the presence of interhemispheric differences and stimulus specificity within the cerebral activation circuit. Finally, based on activation frequencies, cortical maps of external and medial views for both hemispheres were generated to compare the two stimulus types.

**Fig. 2:**
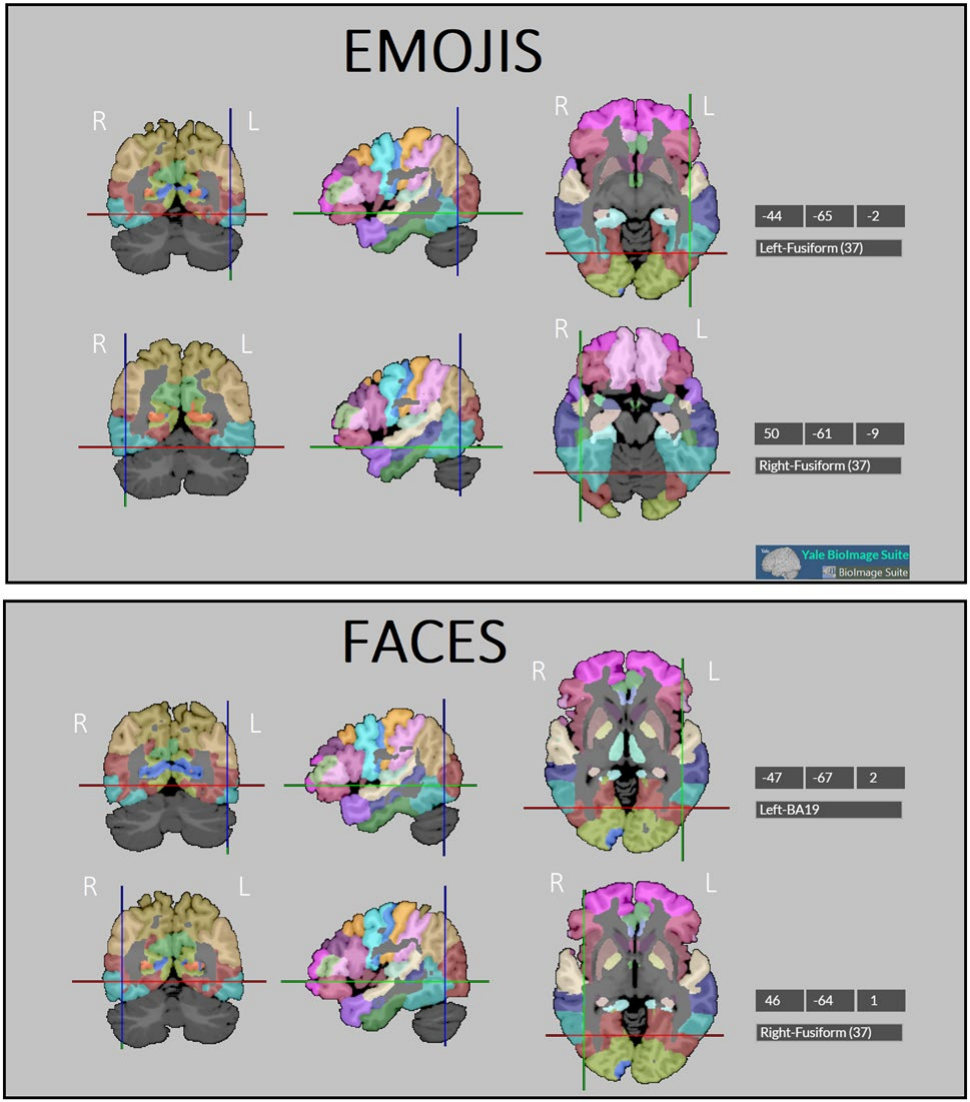
Localization of the most prominent N170 sources for the recognition of emoji **(**top panel) vs. facial expressions (bottom panel). The images depict the average Talairach coordinates of activation, overlaid on a segmented structural brain atlas highlighting Brodmann areas. The colored crosshairs indicate the axial (red), coronal (green), and sagittal (blue) planes. Specific coordinates of peak sources are reported in the adjacent tables. Source localization was performed using the Yale BioImage Suite

Figure 2 illustrates the average Talairach coordinates (X, Y, and Z) of the most prominent left and right N170 sources identified within the regions of interest encompassing BA18, BA19, BA21, and BA37, for emoji and face processing respectively. Source localization was performed using the *Yale BioImage Suite* Version: 1.1.0a9 (2019/03/20). Bilateral Fusiform BA37 sources were identified for emoji processing, while face processing engaged the left occipital face (BA19) area and the right face fusiform area. An ANOVA was applied to dipole strength of individual N170 occipito-temporal sources over the left and right hemisphere, which revealed an effect of stimulus type [F (1,24) = 16.9; *p* < 0.0004; ε = 1; η^2^ = 0.41]. The data indicated stronger signals elicited by emojis (5.29 nA, SE = 0.68) than faces (3.28 nA, SE = 0.40) in the 150–190 latency range. No hemispheric asymmetry was found in the overall strength of BA18, BA19, BA21, and BA37 N170 sources.

**Fig. 3:**
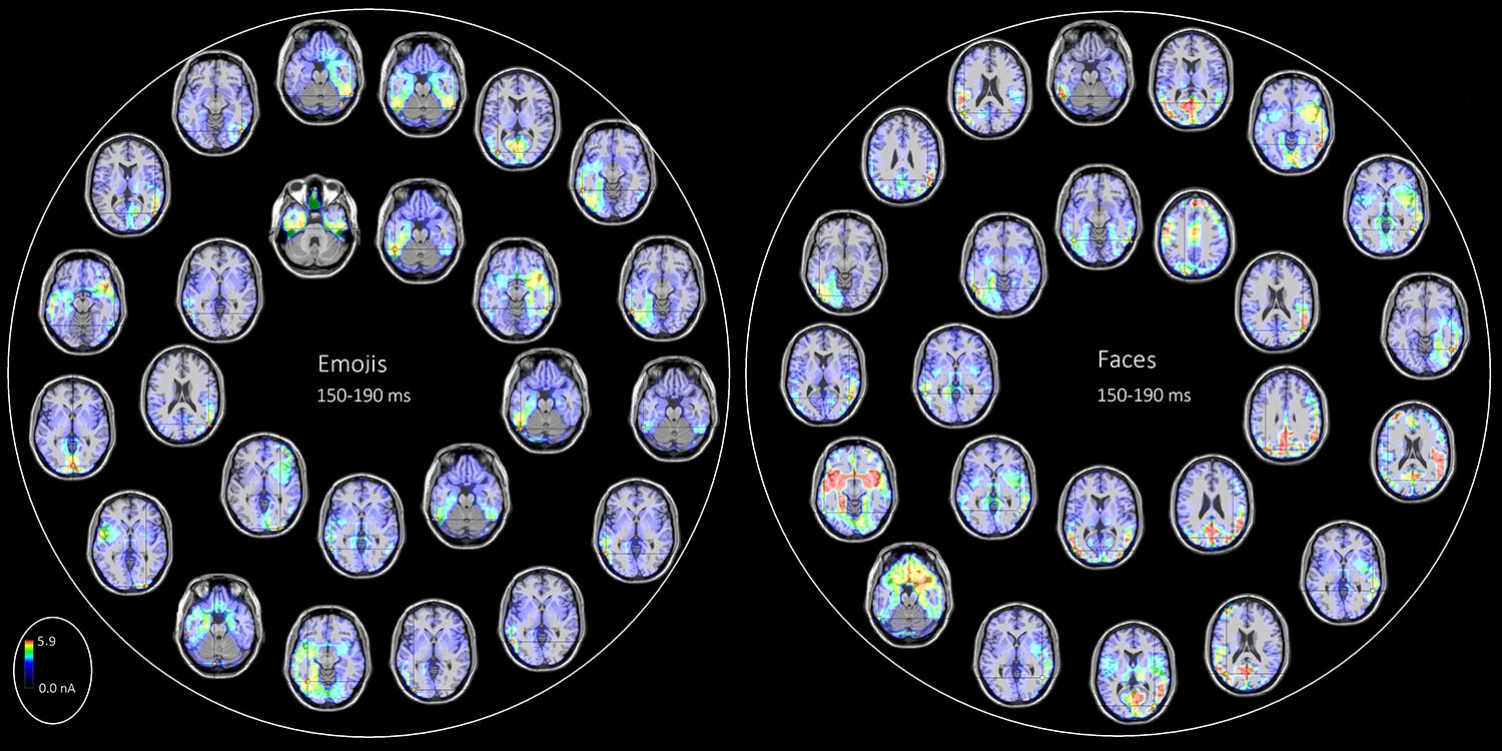
Axial views of individual swLORETA source reconstructions (*N* = 50) of surface potentials performed during perceptual processing of emoji (left) and facial (right) expressions in N170 latency range (150–190 ms). The various colours represent differences in the magnitude of the electromagnetic signal (nA). The electromagnetic dipoles appear as arrows and indicate the position, orientation and magnitude of the dipole modelling solution applied to the ERP waveform in the specific time window

**Fig. 4:**
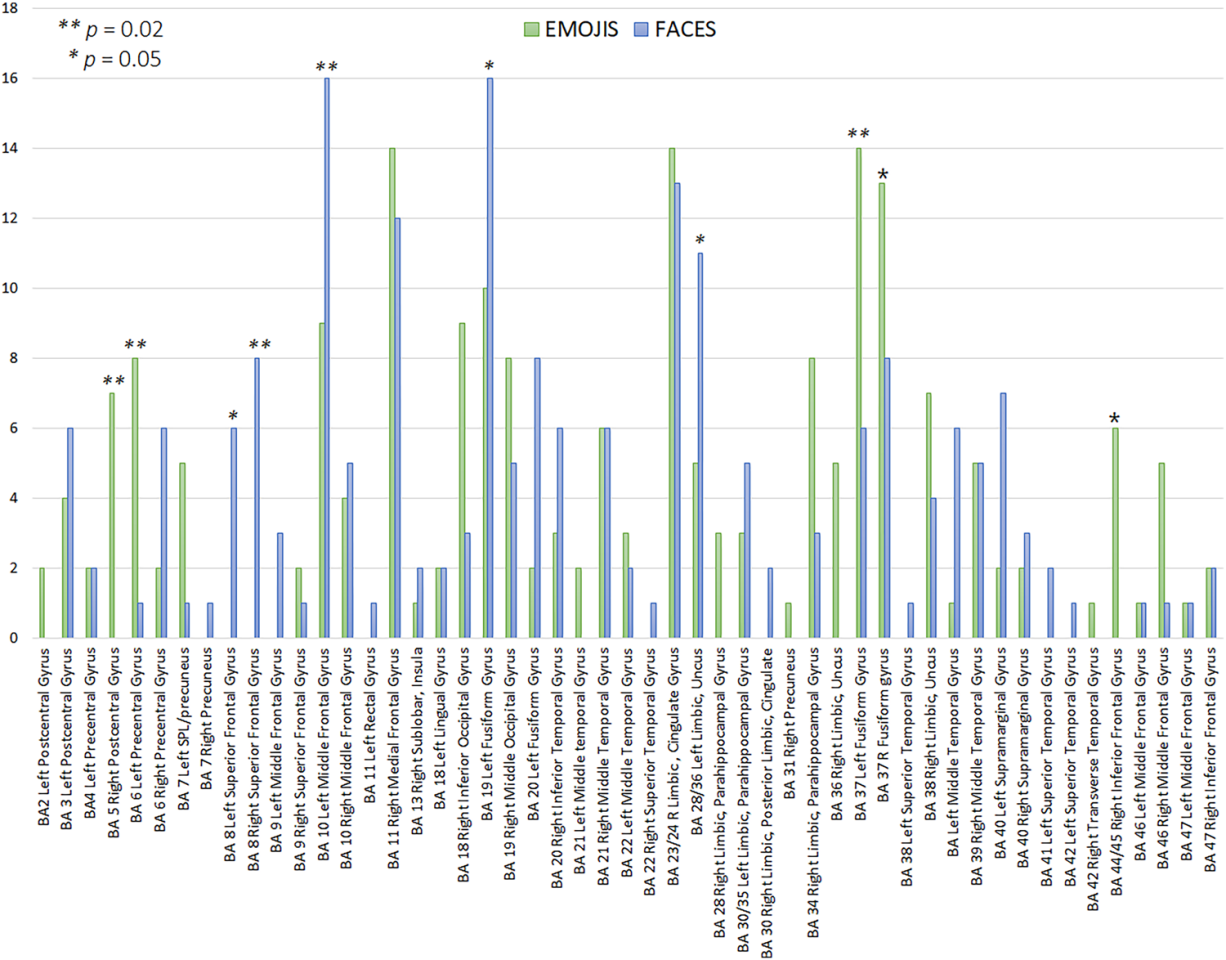
Frequency of significant activation across brain regions of interest (ROIs) during emoji and face expression recognition. The bar chart illustrates the number of times each ROI exhibited significant activation across participants (*N* = 25 per condition) during the processing of emojis (green bars) and faces (blue bars). Statistical significance (between class differences) is indicated by asterisks (*p = 0.05, **p = 0.02). The x-axis lists the BAs and their corresponding anatomical regions, while the y-axis represents the frequency of significant activation

Figure 3 presents axial sections of individual source reconstructions for the face-specific N170 response to congruent emojis (left) and faces (right). Despite the consistent involvement of face-related brain regions, different individuals exhibited variable neural circuit activations. To quantify the extent to which a given brain area (associated with a specific Brodmann Area, BA) was active during emoji vs. face processing, a cumulative analysis of reported activations was conducted. The results of this analysis are displayed in Fig. 4. The bar chart illustrates the frequency of significant activation across regions of interest (ROIs) during emoji and face processing. Results indicate that different brain areas were engaged depending on the stimulus type, with varying degrees of activation across participants (*N* = 25 per condition). Both emoji and face processing recruited overlapping but distinct neural circuits, with some regions showing higher activation frequencies for one stimulus type over the other. The x-axis represents the BAs and their corresponding anatomical regions, while the y-axis denotes the frequency of significant activation across participants. T tests for independent samples were performed on ROIs showing the largest inter-individual difference (≥ 5 units) across conditions (namely: R Postcentral Gyrus, L Precentral Gyrus, L Superior Frontal Gyrus, R Superior Frontal Gyrus, L Middle Frontal Gyrus, R Inferior Occipital Gyrus, L Fusiform Gyrus, L Fusiform Gyrus, L Uncus, R Parahippocampal Gyrus, R Uncus, L Fusiform Gyrus, R Fusiform Gyrus, L Middle Temporal Gyrus, L Supramarginal Gyrus, R Inferior Frontal Gyrus). Statistical significance, marked by asterisks (*p = 0.05, **p = 0.02), highlights key differences in activation between emoji and face processing. All effects remained significant after FDR correction (Benjamini–Hochberg, q < 0.05). These findings suggest that face-related brain regions are consistently engaged, but the extent and specificity of activation vary depending on the stimulus type and individual differences.

Both face and emoji processing recruited occipito-temporal areas traditionally associated with visual and facial recognition, including, BAs 18 and 19 that are key areas for early visual processing, as well as BA 21 and BA 37 (middle and inferior temporal gyri) that are linked to higher-level visual recognition, including face perception. Both stimuli also stimulated the activation of BA 6 (premotor cortex), which may reflect preparatory motor responses to stimuli, linked to task requirements. Very relevant in the emotional processing of face and emoji expression were limbic structures (BA 28, BA 30, BA 34, BA 36) also found active for both stimuli class, implicated in emotion processing and memory integration. These findings suggest that both faces and emojis engaged the core visual and face-processing network, though to different extents.

As for differences across classes, i.e. unique activation patterns for each stimulus type, it was found a stronger activation for faces (blue bars in Fig. 4) for the left fusiform gyrus (BA 19) of the occipital cortex, which might indicate a greater involvement in early-stage visual feature extraction for face details. The left uncus (BA28/36) was significantly more active during face than emoji processing. These results indicate that face perception engages a more specialized, dedicated neural network, particularly in occipito-temporal areas involved in holistic face processing. The left and right superior frontal gyri (BA8) were uniquely active during facial expression and not emoji processing. The area that showed the stronger difference across stimulus type was however the left medial prefrontal cortex (BA 10), supporting mentalizing and theory of mind, that was disproportionally more active during processing of faces of real persons than facial emojis.

**Fig. 5:**
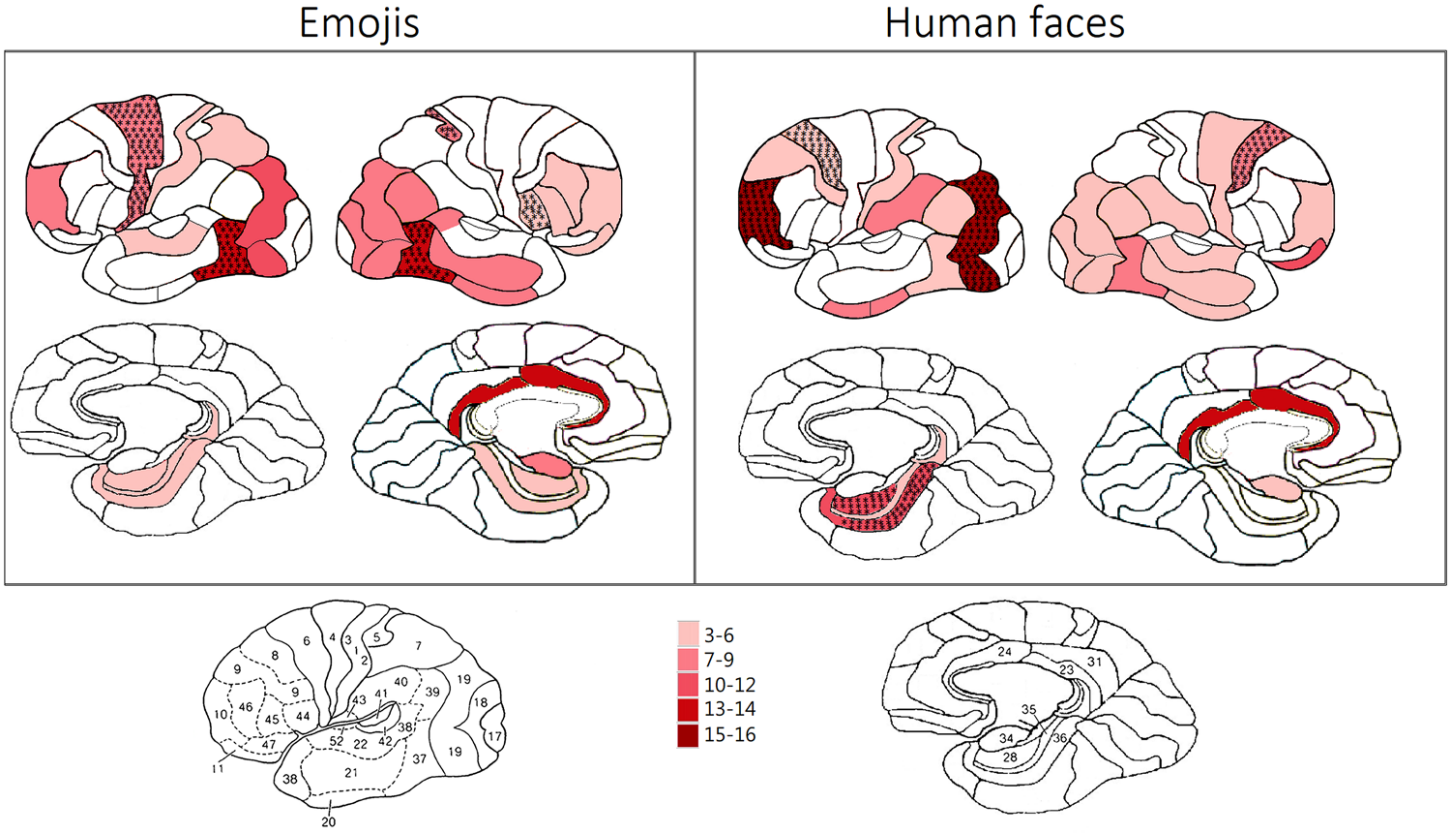
Brain activation patterns for emoji and human facial expression recognition. The left panel illustrates cortical regions activated in response to emojis, while the right panel shows activation for human faces. The color scale represents the number of participants (out of a total sample) exhibiting activation in each region, with darker shades indicating higher consistency across subjects. Hatched regions highlight areas of significant overlap. The bottom row displays medial views of the brain, and the lower inset provides Brodmann area annotations for reference

On the opposite side, a stronger activation for Emojis (green bars in Fig. 4) was found over the right post-central gyrus (BA 5) and the left Precentral gyrus (BA 6), suggesting increased sensorimotor integration (potentially due to learned associations with digital communication). Again, the left and right fusiform gyri BA 37 showed a greater activation for emojis, possibly reflecting reading processing and symbol interpretation. Finally, the right inferior frontal gyrus IGF (BA44/45) was uniquely active during emoji and not face processing (as also illustrated in Fig. 5). These findings imply that emoji perception relies more on sensorimotor and semantic networks, possibly reflecting their role as symbolic representations of emotions rather than naturally occurring facial expressions.

**Fig. 6:**
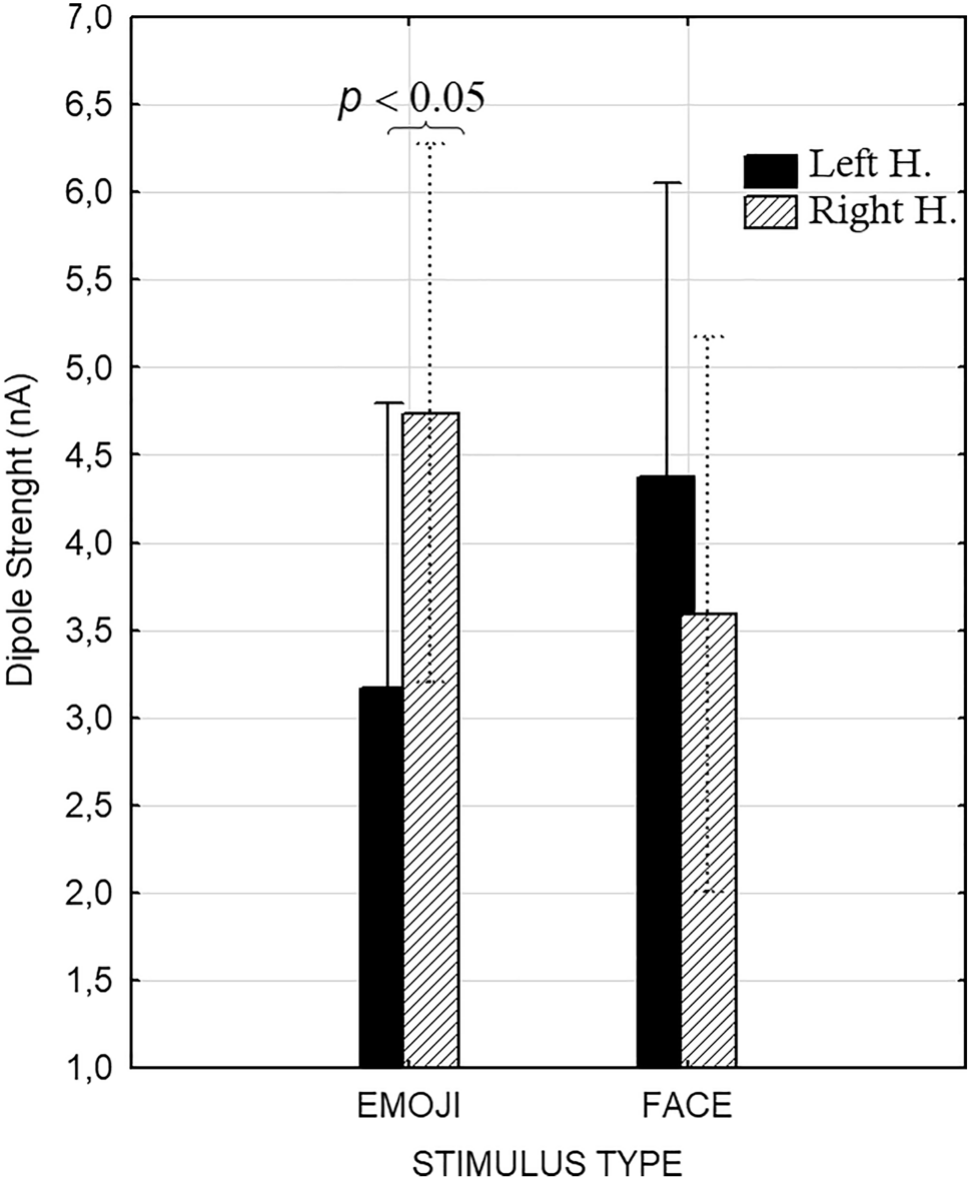
Hemispheric asymmetry in the frequency of ROI activations across brain areas in the two perceptual conditions

A repeated measures ANOVA was performed on the same set of data for extracting a general index of hemispheric asymmetry recruitment across experimental conditions and individual. The ANOVA yielded the significant interaction of type of stimulation (Emoji vs. facial) x Cerebral hemisphere (Left, right) [F(1,49) = 35.3; *p* < 0.028; η^2^ = 0.10]. The results showed the significant interaction of stimulus type x hemisphere (see Fig. 6). Post hoc comparisons that during emoji processing right brain areas were more frequently active, whereas there was no significant hemispheric difference for face processing.

## Discussion

In this study, we conducted a participant-by-participant analysis of brain activations using swLORETA source reconstruction in a sample of 50 healthy, young, right-handed individuals. Participants performed a facial expression recognition task involving six distinct expressions, presented either as real human faces (male or female, from eight different identities) or as emojis in eight different stylistic variations—an entirely novel approach in the literature. The analysis of neural circuits at the individual level allows for a more precise examination of the underlying mechanisms, which are often concealed or diminished by group averaging. In group-level analyses, activations observed in only 6, 10, or even 15 out of 25 participants might be eliminated, whereas this type of analysis clearly highlighted them and accounted for them statistically.

Overall, accuracy in emotional recognition of facial expressions was higher for emojis (hits = 92.7%) than faces (hits = 82.4%). This advantage of emojis over faces in terms of accuracy and response speed was strongly consistent with the P300 amplitude data reported in Dalle Nogare and Proverbio (2023), which showed larger congruence effect (i.e., larger amplitudes for congruent patterns than for incongruent patterns) for emojis than for faces. This seems to suggest how emoji, due to their schematic nature, may be easier to recognize than faces, as also reported by previous studies especially for negative emotions such as fear (Fischer and Herbert 2021) anger or disgust (Cherbonnier and Michinov 2021). Consistently, in Dalle Nogare and Proverbio (2023) both N400 and P300 were considerably later in response to faces than emojis, while N170 was larger to emoji than faces in Gantiva et al. (2019).

In this study, the higher activation observed for human faces in anterior brain regions (mainly BA 10 and BA 8, but also BA 9) is likely indicative of cognitive processes involved in the attribution of thoughts, intentions, and beliefs—core mechanisms of Theory of Mind (ToM). Additionally, bilateral superior forntal gyri BA 8 (also known as frontal eye fields) is involved in the processing of eye movement and attentional allocation (Vernet et al. 2014). This suggests that the human brain engages more extensively in mentalizing and social inference when processing faces compared to emojis, reinforcing the idea that facial stimuli carry a greater biological and social significance. Notably, while N170 predominantly indexes early structural encoding; nonetheless, evidence of early prefrontal activations in socially salient contexts (e.g., Proverbio & Galli, 2016) points to an immediate recruitment of affective or proto-mentalizing processes. Moreover, face processing was uniquely associated with the activation of the left uncus—a component of the limbic system directly connected to the amygdala, parahippocampal region, and temporal cortex—which plays a crucial role in emotion processing and volitional emotional expressions (Iwase et al. 2002), as well as in the recognition of emotional facial expressions (Szaflarski et al. 2018; Proverbio et al. 2014). On the other side, the stronger activation for emoji in the bilateral temporal cortex, right inferior frontal gyrus, and superior parietal cortex may reflect a greater reliance on symbolic processing and abstract visual perception. This pattern aligns with findings on numerical cognition, where symbolic representations such as numerals recruit a network spanning the inferior temporal, parietal, and frontal cortices. Specifically, the posterior inferior temporal gyrus has been implicated in numeral recognition (Yeo et al. 2020), and the inferior temporal numeral area (ITNA) exhibits distinct connectivity patterns: the left ITNA is structurally connected to Broca’s area, suggesting a role in verbal-symbolic associations, while the right ITNA is more strongly connected to the ipsilateral inferior parietal cortex and functionally coupled with the bilateral intraparietal sulcus (IPS) (Conrad et al. 2023). Given that the IPS and posterior superior parietal lobule (PSPL) are crucial for mental arithmetic and number reading (Proverbio & Carminati, 2019; Andres et al. 2012), the engagement of similar temporo/parietal and fronto/parietal networks during emoji expression recognition suggests that both numerals and emojis may rely on shared neural mechanisms for interpreting abstract visual symbols. This highlights the shared role of these brain regions in processing abstract symbols, whether numerical or visual (such as emojis). In an interesting study with a similar paradigm by Chatzichristos et al. (2020) it was found that emotional incongruity among word + emoji combinations activated the Broca’s area (BA44 and BA45) in both hemispheres, and the inferior prefrontal cortex (BA47), compared to congruent combinations, which strongly fits with the present pattern of results. Recent evidence further supports the idea that emojis can modulate affective TOM processes (aTOM). Zhong et al. (2025) reported that text–emoji valence congruency significantly influenced neural markers of emotional state attribution, with larger N200 and late components for incongruent combinations—reflecting greater cognitive effort during affective inference. These findings complement the present focus on early perceptual encoding (N170) by showing that emojis also modulate later stages of affective ToM processing. Importantly, whereas Zhong et al. (2025) required explicit affective mental-state attribution—thereby engaging higher-level ToM computations reflected in N200 and later cognitive components—our paradigm minimized explicit ToM demands and instead targeted earlier perceptual–structural encoding of socio-emotional symbols. Moreover, while their study manipulated broad valence categories, the present design employed six discrete basic emotions, offering a more fine-grained view of emotion-specific symbolic processing.

Overall, the findings suggest that interpreting emojis engages distinct cognitive strategies compared to natural facial expressions. The generally higher amplitude of brain signals during emoji-based emotional recognition indicates that, at this early processing stage, schematic stimuli (emojis) elicit a stronger neural response, aligning with evidence of larger N170 amplitudes (e.g., Gantiva et al. 2019). This effect may be attributable to the simplified and exaggerated features of emojis, which facilitate the classification of affective content. This hypothesis is further supported by behavioral findings showing that, on average, reaction times (RTs) for emoji recognition were 73 ms faster than for facial expressions, and accuracy was higher for emojis (92.7%) than for facial expressions (87.35%) (Dalle Nogare and Proverbio 2023). The hemispheric asymmetry observed in overall cerebral activation for emoji recognition—but not for facial expression recognition—further supports the notion that these two types of social cues may be processed in fundamentally different ways. Notably, regions more specifically associated with facial expression processing included left-hemisphere structures such as the left uncus, left fusiform gyrus, left medial frontal gyrus, and left superior frontal gyrus. Research indicates that the medial prefrontal cortex (mPFC), which includes the left medial frontal gyrus, is strongly associated with abstract social cognition processes such as mentalizing and theory of mind. Notably, mentalizing activates much of the mPFC, with dorsal regions appearing more selective for information about others, while anterior regions may be more selective for self-related information. Several studies have demonstrated the involvement of the left medial prefrontal cortex (mPFC) in abstract social cognition processes such as mentalizing and theory of mind. For instance, a meta-analysis by Denny et al. (2012) examined 107 functional neuroimaging studies and found that tasks involving mentalizing were more associated with activation in the left medial BA 10, especially if related to Self (see also Depalma and Proverbio 2024).

All in all, the results indicate that facial expression comprehension relies more on socio-affective and face-processing networks, while emoji perception engages circuits involved in symbolic interpretation, semantic processing, and sensorimotor integration. While both faces and emojis activated core visual processing areas, face perception was more reliant on limbic structures such as the left uncus (engaged in instinctual emotional expression and recognition) but especially medial and superior frontal areas (key structures for TOM and mentalizing). On the other hand, emoji expression processing recruited more sensorimotor and linguistic regions (BA 5, 6, 44, 45). This suggests that while emojis mimic facial expressions, their neural processing differs significantly, involving mechanisms more akin to language and symbolic recognition. Faces are processed as natural social stimuli, involving a human relation and a mind attribution while emojis may be interpreted as learned symbols within digital communication. We also note that, unlike emoji, human faces exhibit both individual variability and clear sexual dimorphism in facial morphology: this unexplored factor could further contribute to amplifying the differences between categories. It should be noted that the present findings pertain specifically to neural mechanisms engaged during emotion recognition under congruent (word–stimulus matching) conditions. Therefore, they reflect differences in early perceptual and recognition networks rather than processes associated with semantic incongruence or higher-order mentalizing demands.

### Conclusions, Limitations, and Future Directions

The distinction between human and non-human manifests in divergent neural responses, particularly in the realms of affective comprehension, the construction and attribution of a mind, and the recognition of another human presence—none of which occur in response to emojis.

A potential limitation of the study is the sample size, which, while adequate, could be further improved. Additionally, the analysis was restricted to brain activity at the level of the N170 (150–190 ms). Future research should extend the investigation to other temporal windows to enhance our understanding of the complex neural systems involved in processing social stimuli. Another potential limitation of the present design is that face and emoji processing were examined within a word-primed paradigm. Therefore, some of the observed differences might reflect distinct interactions between each stimulus type and verbal priming (e.g., semantic integration), rather than intrinsic differences in face versus emoji processing. Future studies should address this potential confound by employing nonverbal or cross-modal paradigms to disentangle modality-specific effects from priming-related influences.

## Data Availability

The authors confirm that the data supporting the findings of this study are available within the article. Other information are available on request from the corresponding author. The data are not publicly available due to privacy or ethical restrictions.
